# Emergency Department Visits for Sports- and Recreation-Related Traumatic Brain Injuries Among Children — United States, 2010–2016

**DOI:** 10.15585/mmwr.mm6810a2

**Published:** 2019-03-15

**Authors:** Kelly Sarmiento, Karen E. Thomas, Jill Daugherty, Dana Waltzman, Juliet K. Haarbauer-Krupa, Alexis B. Peterson, Tadesse Haileyesus, Matthew J. Breiding

**Affiliations:** ^1^Division of Unintentional Injury Prevention, National Center for Injury Prevention and Control, CDC; ^2^Division of Analysis, Research, and Practice Integration, National Center for Injury Prevention and Control, CDC.

Traumatic brain injuries (TBIs), including concussions, are at the forefront of public concern about athletic injuries sustained by children. Caused by an impact to the head or body, a TBI can lead to emotional, physiologic, and cognitive sequelae in children (*1*). Physiologic factors (such as a child’s developing nervous system and thinner cranial bones) might place children at increased risk for TBI (*2*,*3*). A previous study demonstrated that 70% of emergency department (ED) visits for sports- and recreation-related TBIs (SRR-TBIs) were among children (*4*). Because surveillance data can help develop prevention efforts, CDC analyzed data from the National Electronic Injury Surveillance System–All Injury Program (NEISS-AIP)* by examining SRR-TBI ED visits during 2010–2016. An average of 283,000 children aged <18 years sought care in EDs each year for SRR-TBIs, with overall rates leveling off in recent years. The highest rates were among males and children aged 10–14 and 15–17 years. TBIs sustained in contact sports accounted for approximately 45% of all SRR-TBI ED visits. Activities associated with the highest number of ED visits were football, bicycling, basketball, playground activities, and soccer. Limiting player-to-player contact and rule changes that reduce risk for collisions are critical to preventing TBI in contact and limited-contact sports. If a TBI does occur, effective diagnosis and management can promote positive health outcomes among children.

NEISS-AIP is operated by the U.S. Consumer Product Safety Commission and contains data on initial visits for all injuries in patients treated in U.S. hospital EDs. NEISS-AIP data are drawn from a nationally representative subsample of 66 of 100 NEISS hospitals that were selected as a stratified probability sample of hospitals in the United States and its territories; each hospital has a minimum of six beds and a 24-hour ED (*5*). NEISS-AIP provides data on approximately 500,000 injury-related visits each year.

For this analysis, SRR-TBIs included those TBIs among children aged <18 years that occurred during organized and unorganized SRR activities. Each case was classified into mutually exclusive SRR categories based on an algorithm that uses the consumer products involved and the description of the incident from the medical record. Persons with injuries were classified as having a TBI if the primary body part injured was the head and the principal diagnosis was concussion or internal organ injury. Type of activity (i.e., contact sport, limited-contact sport, noncontact sport, or recreation) was determined based on classifications from previous studies.^†^ SRR-TBI cases were excluded if the injury was violence-related or if the person was dead on arrival or died in the ED. Methodology for coding and classifying data matched that of a previously published report (*6*). The Joinpoint Regression Program (version 4.2.0; National Cancer Institute) was used to test time trends.

The overall rate of SRR-TBI ED visits did not change significantly from 2010 (354.7 visits per 100,000 children) to 2016 (371.0); however, there were differences by sex (Table 1). Throughout the study period, the number and rate of SRR-TBI ED visits by males were higher than were those among females. The rate of SRR-TBI ED visits in males significantly increased from 2010 (486.6) to 2012 (559.1) and significantly decreased from 2012 to 2016 (482.7). However, the rate in females significantly increased from 216.5 per 100,000 children in 2010 to 254.3 in 2016. During all 7 years, children aged 10–14 and 15–17 years had higher rates of ED visits than did children in all younger age groups.

**TABLE 1:** Estimated annual number and rate* of emergency department visits for all nonfatal traumatic brain injuries (TBIs) related to sports and recreation activities among persons aged <18 years, by selected characteristics — National Electronic Injury Surveillance System–All Injury Program, United States, 2010–2016

Characteristic	2010		2011		2012		2013		2014		2015		2016	
	No.^†^	Rate (95% CI)	No.^†^	Rate (95% CI)	No.^†^	Rate (95% CI)	No.^†^	Rate (95% CI)	No.^†^	Rate (95% CI)	No.^†^	Rate (95% CI)	No.^†^	Rate (95% CI)
**Age group (yrs)**														
0–4	24,161	119.6 (83.0–156.2)	23,485	116.7 (74.0–159.4)	23,957	119.9 (84.3–155.5)	20,553	103.6 (75.2–132.0)	20,930	105.3 (75.6–135.1)	20,983	105.4 (72.6–138.1)	23,232	116.6 (72.7–160.5)
5–9	52,536	258.2 (186.0–330.3)	55,800	274.4 (206.4–342.5)	61,011	298.1 (226.5–369.7)	59,690	290.2 (224.7–355.7)	56,837	277.0 (202.6–351.5)	62,175	303.6 (212.1–395.2)	58,899	288.3 (184.0–392.6)
10–14	105,736	511.4 (386.0–636.7)	109,112	526.7 (389.4–664.1)	128,672	622.5 (460.0–784.9)	125,588	608.1 (451.3–764.8)	122,359	592.0 (459.0–724.9)	125,446	608.7 (461.5–755.8)	113,664	551.0 (400.6–701.4)
15–17	80,686	622.9 (471.5–774.2)	84,836	665.9 (512.6–819.1)	89,327	709.7 (525.3–894.2)	89,466	715.4 (521.4–909.5)	89,355	714.0 (530.5–897.4)	78,655	622.9(479.1–766.8)	77,477	610.9(431.3–790.4)
**Sex**														
Male^§,¶^	184,651	486.6 (366.7–606.6)	191,341	506.4 (379.0–633.8)	210,569	559.1 (418.0–700.3)	202,575	539.0 (411.9–666.1)	198,678	528.7 (403.4–654.0)	190,943	507.7 (384.0–631.4)	181,623	482.7 (345.7–619.8)
Female**	78,468	216.5 (162.3–270.8)	81,891	226.7 (172.0–281.4)	92,398	256.4 (191.3–321.5)	92,723	257.6 (183.5–331.7)	90,803	252.3 (190.2–314.4)	96,317	267.4 (198.3–336.5)	91,649	254.3 (174.2–334.5)
**Total**	**263,118**	**354.7 (267.7–441.6)**	**273,232**	**369.7 (278.7–460.7)**	**302,966**	**411.1 (308.1–514.0)**	**295,297**	**401.4 (301.4–501.3)**	**289,481**	**393.5 (300.1–486.9)**	**287,260**	**390.1 (294.2–486.1)**	**273,272**	**371.0 (262.2–479.8)**

**Abbreviation:** CI = confidence interval.

* Per 100,000 population.

^†^ Numbers might not sum to totals because of rounding.

^§^ Rate significantly increased from 2010 to 2012.

^¶^ Rate significantly decreased from 2012 to 2016.

** Rate significantly increased from 2010 to 2016.

From 2010 to 2016, contact sports were associated with a higher number of TBI-related ED visits by males (99,784) than were limited contact sports (29,080), noncontact sports (44,848), and recreational activities (20,628) (Table 2). Among females, contact sports (27,180) and limited contact sports (27,343) contributed to a similar number of SRR-TBI-related ED visits. Football contributed to more ED visits (52,088) among males than did any other sport. Soccer (11,670) and playground activities (11,255) contributed to more TBI-related ED visits among females than did all other activities.

**TABLE 2:** Average annual estimates of emergency department visits for all nonfatal traumatic brain injuries (TBIs) related to sports and recreation activities among persons aged <18 years, by type of activity — National Electronic Injury Surveillance System–All Injury Program, United States, 2010–2016

Activity	No* (95% CI)		
	Overall	Males	Females
**Contact sports**	126,964 (96,564–157,364)	99,784 (76,521–123,047)	27,180 (19,449–34,911)
Football	53,657 (42,998–64,316)	52,088 (41,640–62,536)	1,570 (1,197–1,943)
Basketball	29,675 (22,497–36,853)	19,057 (14,303–23,811)	10,617 (8,074–13,160)
Soccer	23,847 (15,107–32,587)	12,177 (7,972–16,382)	11,670 (7,011–16,329)
Hockey^†^	8,110^§^ (2,210–14,010)^§^	6,697^§^ (1,271–12,123)^§^	1,412 (642–2,182)
Combative sports^¶^	6,798 (4,898–8,698)	6,372 (4,539–8,205)	426 (274–578)
Miscellaneous contact ball games**	4,877 (3,051–6,703)	3,392 (2,076–4,708)	1,485 (916–2,054)
**Limited contact sports**	56,423 (42,674–70,172)	29,080 (21,405–36,755)	27,343 (20,782–33,904)
Baseball	14,208 (10,501–17,915)	11,888 (8,671–15,105)	2,320 (1,749–2,891)
Gymnastics^††^	8,008 (5,609–10,407)	723 (482–964)	7,284 (5,049–9,519)
Skateboarding	6,857 (3,714–10,000)	5,618 (2,881–8,355)	1,239 (728–1,750)
Softball	5,675 (3,898–7,452)	521 (309–733)	5,155 (3,532–6,778)
Trampolining	4,906 (3,276–6,536)	2,976 (2,034–3,918)	1,930 (1,174–2,686)
Horseback riding	3,427 (2,222–4,632)	605^§^ (234–976)^§^	2,822 (1,897–3,747)
Volleyball	3,268 (2,549–3,987)	439 (262–616)	2,829 (2,217–3,441)
Ice skating	2,227 (1,297–3,157)	1,180 (696–1,664)	1,047 (546–1,548)
In-line/Roller skating	2,041 (1,328–2,754)	1,048 (630–1,466)	993 (616–1,370)
Other limited contact sports^§§^	5,806^§^ (2,280–9,332)^§^	4,081^§^ (1,566–6,596)^§^	1,725^§^ (674–2,776)^§^
**Noncontact sports**	68,684 (52,391–84,977)	44,848 (34,335–55,361)	23,836 (17,995–29,677)
Playground	27,350 (19,582–35,118)	16,095 (11,697–20,493)	11,255 (7,831–14,679)
Bicycling	25,955 (19,985–31,925)	19,880 (15,333–24,427)	6,075 (4,539–7,611)
Swimming	6,796 (5,131–8,461)	3,754 (2,685–4,823)	3,042 (2,303–3,781)
Exercise	5,030 (3,820–6,240)	3,054 (2,294–3,814)	1,976 (1,380–2,572)
Golf^¶¶^	1,748 (1,126–2,370)	1,084 (712–1,456)	665 (352–978)
Track and field	1,074 (683–1,465)	571 (313–829)	503 (300–706)
Racquet sports***	570 (342–798)	298 (151–445)	272 (147–397)
Bowling	160^§^ (71–249)^§^	113^§^ (40–186)^§^	47^§^ (3–91)^§^
**Recreation**	31,447 (24,905–37,989)	20,628 (16,543–24,713)	10,819 (8,256–13,382)
Scooter riding	5,711 (3,903–7,519)	3,811 (2,539–5,083)	1,900 (1,282–2,518)
All-terrain vehicle riding	4,702 (2,339–7,065)	3,046 (1,583–4,509)	1,656 (723–2,589)
Amusement attractions^†††^	2,989 (2,043–3,935)	1,633 (1,136–2,130)	1,356 (817–1,895)
Tobogganing/Sledding	2,988 (2,079–3,897)	1,793 (1,215–2,371)	1,194 (796–1,592)
Moped/Dirt bike riding^§§§^	2,921 (2,161–3,681)	2,536 (1,880–3,192)	386 (227–545)
Other recreation^¶¶¶^	1,323 (824–1,822)	800 (458–1,142)	523 (303–743)
Miscellaneous recreation ball games****	4,090 (3,017–5,163)	2,711 (2,045–3,377)	1,379 (876–1,882)
Other specified^††††^	6,723 (5,098–8,348)	4,298 (3,175–5,421)	2,425 (1,799–3,051)
**Total**	**283,518 (218,675–348,361)**	**194,340 (150,416–238,264)**	**89,178 (68,133–110,223)**

**Abbreviation:** CI = confidence interval.

* Estimates might not sum to totals because of rounding.

^†^ Includes ice hockey, field hockey, roller hockey, and street hockey.

^§^ Estimates were identified as unstable if the number of sample cases was <20, the weighted estimate was <1,200, or the coefficient of variation was >30.

^¶^ Includes boxing, wrestling, martial arts, and fencing.

** Includes lacrosse, rugby, and handball.

^††^ Includes cheerleading and dancing.

^§§^ Includes snow skiing, snowboarding, water skiing, and surfing.

^¶¶^ Includes injuries related to golf carts.

*** Includes tennis, badminton, and squash.

^†††^ Includes rides and water slides (not swimming pool slides).

^§§§^ Includes other two-wheeled, powered, off-road vehicles and dune buggies.

^¶¶¶^ Includes nonpowder/BB guns, go carts, personal watercraft, snowmobiling, camping, fishing, and billiards.

**** Includes tetherball, kick ball, and dodgeball.

^††††^ Includes gym/physical education class, archery, darts, curling, and mountain climbing.

SRR-activities associated with the highest percentage of ED visits varied by age group and sex (Table 3). Football was associated with 26.8% of all SRR-TBI ED visits for males aged 0–17 years. Among males aged <5 years and 5–9 years, playground activities accounted for the most ED visits (38.2% and 19.6%, respectively). Among all females aged 0–17 years, soccer, playground activities, and basketball were the most common causes of SRR-TBI ED visits, contributing to 13.1%, 12.6%, and 11.9% of all SRR-TBI-related ED visits, respectively. Playground activities led to 42.3% of SRR-TBIs visits among females aged <5 years.

**TABLE 3:** Average annual estimates of the five most common activities associated with emergency department visits for nonfatal traumatic brain injuries related to sports or recreation activities, by age group and sex — National Electronic Injury Surveillance System–All Injury Program, United States, 2010–2016

Age group (yrs)	No.* of sport or recreational TBI-related ED visits	No.* (% of all sport or recreational TBI-related ED visits)				
		Sport or recreational activity				
	Total	Football	Basketball	Playground	Bicycle	Soccer
**Males**						
**All 0–17**	**194,340**	**52,088 (26.8)**	**19,057 (9.8)**	**16,095 (8.3)**	**19,880 (10.2)**	**12,177 (6.3)**
<5	14,394	120^†^ (0.8)	431 (3.0)	5,504 (38.2)	2,180 (15.1)	363^†^ (2.5)
5–9	39,673	5,216 (13.1)	2,662 (6.7)	7,792 (19.6)	5,269 (13.3)	1,971 (5.0)
10–14	83,941	27,343 (32.6)	9,635 (11.5)	2,558 (3.0)	7,904 (9.4)	5,524 (6.6)
15–17	56,332	19,408 (34.5)	6,330 (11.2)	240 (0.4)	4,526 (8.0)	4,319 (7.7)
**Females**						
**All 0–17**	**89,178**	**1,570 (1.8)**	**10,617 (11.9)**	**11,255 (12.6)**	**6,075 (6.8)**	**11,670 (13.1)**
<5	8,078	44^†^ (0.5)	141^†^ (1.7)	3,418 (42.3)	861 (10.7)	140^†^ (1.7)
5–9	18,463	155^†^ (0.8)	656 (3.6)	5,628 (30.5)	2,599 (14.1)	784^†^ (4.2)
10–14	34,713	793 (2.3)	4,948 (14.3)	1,886 (5.4)	1,899 (5.5)	5,939 (17.1)
15–17	27,925	577 (2.1)	4,873 (17.5)	324 (1.2)	716 (2.6)	4,806 (17.2)

* Numbers might not sum to totals because of rounding.

^†^ Estimates were identified as unstable if the number of sample cases was <20, the weighted estimate was <1,200, or the coefficient of variation was >30.

## Discussion

Across the 7-year study period, an estimated 2 million children aged <18 years visited an ED because of a TBI sustained during SRR activities. A previous report found a sharp increase from 2006 to 2012 in the rate of SRR-TBI ED visits (*4*). Results from the current study suggest there has been a leveling off of overall SRR-TBI ED visits since the last report and a significant decline for males since 2012. Going forward, surveillance for TBI should explore these changes in the SRR-TBI ED visit trends to help develop ongoing and future prevention strategies. Potential reasons for this decline in males might include successful prevention efforts (e.g., safety-minded rule changes in contact sports), reduced participation in contact sports, or changes in care-seeking behaviors.

In all study years, males had approximately twice the rate of SRR-TBI ED visits as did females, which is consistent with other studies suggesting that males are at higher risk (*4*,*7*). SRR-TBI rates also generally increased with age, with children aged 10–14 and 15–17 years having the highest rates SRR-TBIs. These results are likely associated with greater participation of males and older children in contact sports.

Children participating in any SRR activity are at risk for TBI, and earlier studies found higher rates of TBI in sports in which collisions among athletes are more common, such as in football, soccer, basketball, lacrosse, ice hockey, and wrestling (*7*). Consistent with those studies, this report found that contact sports resulted in nearly twice as many TBI ED visits as did noncontact sports and four times those associated with recreation-related activities. Preparticipation athletic examinations are an important opportunity for health care providers to identify athletes who might be more susceptible to a TBI and prolonged recovery (such as older children/adolescents and persons with a history of previous TBI or intracranial injury, learning difficulties or lower cognitive ability, neurologic or psychiatric disorder, lower socioeconomic status, and family and social stressors) (*8*) and to discuss sports-specific injury prevention strategies. In addition, promoting prevention strategies in sports, including limiting player-to-player contact and rule changes that reduce risk for collisions is critical to preventing TBIs (*8*). Further research on the impact of strict officiating, state policies, and presence of athletic trainers in preventing sports-related TBI might be beneficial (*8*).

CDC published an evidence-based guideline on the diagnosis and management of pediatric mild TBI, including concussion, in 2018 (*1*). Five important recommendations in the CDC Pediatric Mild TBI Guideline include 1) not routinely imaging pediatric patients to diagnose mild TBI; 2) using validated, age-appropriate symptom scales to diagnose mild TBI; 3) assessing for risk factors for prolonged recovery; 4) providing patients with instructions on returning to activity customized to their symptoms; and 5) counseling patients to return gradually to nonsports activities after no more than 2–3 days of rest. To help implement these recommendations, CDC created educational tools that are available at https://www.cdc.gov/HEADSUP.

The findings in this report are subject to at least five limitations. First, injury rates for specific activities could not be calculated because of a lack of national participation and exposure data. Therefore, the estimates cannot be used to calculate the relative risks for TBI associated with any particular SRR activity. Second, NEISS-AIP includes only injuries resulting in visits to hospital EDs. Research suggests that many children with a TBI do not seek care in EDs or do not seek care at all, resulting in a significant underestimate of prevalence (*9*). Third, because NEISS-AIP includes only the principal diagnosis and primary body part recorded during the initial injury visit, some cases for which TBI was a secondary diagnosis (for example, skull fractures, which often have a co-occurring TBI diagnosis) might have been missed. Fourth, NEISS-AIP narrative descriptions do not provide detailed information about injury circumstances (e.g., whether the activity was organized, whether the injury occurred during practice or competition, or whether protective equipment was used). Finally, the available data do not allow for assessment of whether any observed differences in the number of ED visits resulted from a true change in incidence, care-seeking behaviors, or other reasons.

TBIs in sports and recreational activities remain a significant public health problem. Limiting player-to-player contact and rule changes that reduce risk for collisions are critical to preventing TBI in contact and limited-contact sports. Development and testing of evidence-based interventions tailored for individual noncontact sports and recreation activities are warranted to ensure that children can stay healthy and active.

**Summary**

**What is already known about this topic?**

Traumatic brain injury (TBI), a common injury among young athletes, can lead to short- or long-term emotional, physiologic, and cognitive sequelae.

**What is added by this report?**

An estimated, 283,000 children seek care in U.S. emergency departments each year for a sports- or recreation-related TBI. TBIs sustained in contact sports account for approximately 45% of these visits. Football, bicycling, basketball, playground activities, and soccer account for the highest number of emergency department visits.

**What are the implications for public health practice?**

Primary prevention efforts tailored to specific sports and recreation-related activities are critical to reducing the risk for childhood TBI. Effective diagnosis and management of a TBI can promote positive health outcomes among children.

## Footnotes

* https://cpsc.gov/Research--Statistics/NEISS-Injury-Data.

† https://fpnotebook.com/sports/Exam/SprtsCntctLvls.htm.
